# Lessons Learned from a Community-led, Pilot Teletherapy Group for Older Women Living with Depression and HIV

**DOI:** 10.1007/s10461-024-04468-y

**Published:** 2024-09-02

**Authors:** Aaron S. Breslow, Michelle Lopez, Barbara Warren, Jules Levin, Anjali Sharma, Dana Watnick, Ginette Sims, Elizabeth Cavic, Obioesio Bassey, Marla R. Fisher, Laurie J. Bauman

**Affiliations:** 1 PRIME Center for Health Equity, Psychiatry Research Institute at Montefiore Einstein, Department of Psychiatry and Behavioral Sciences, Albert Einstein College of Medicine, Bronx, NY USA; 2 Department of Psychiatry & Behavioral Sciences, Montefiore Medical Center, Bronx, NY USA; 3 Einstein-Rockefeller-CUNY Center for AIDS Research, Albert Einstein College of Medicine, Bronx, NY USA; 4 PRIME Center for Health Equity, Department of Psychiatry and Behavioral Sciences, Albert Einstein College of Medicine, 1300 Morris Park Avenue, Van Etten 4A-47, Bronx, NY 10461 USA; 5 LGBT Programs and Policies, Office for Diversity and Inclusion, Mount Sinai Health System, New York, NY USA; 6 Icahn School of Medicine at Mount Sinai, New York, NY USA; 7 National AIDS Treatment Advocacy Project, New York, NY USA; 8 Divisions of General Internal Medicine and Infectious Diseases, Albert Einstein College of Medicine, Bronx, NY USA; 9 Mount Sinai Morningside/West, Icahn School of Medicine at Mount Sinai, New York, NY USA

**Keywords:** HIV/AIDS, Psychotherapy, Group teletherapy, Psychosocial well-being

## Abstract

Older women with HIV face challenges to their quality of life, including neurocognitive decline, early-onset menopause, and chronic health issues. Chief among these concerns is depression, the most common psychiatric comorbidity among people living with HIV, with rates twice as high among women as men. However, tailored interventions among older women living with HIV and depression are lacking. Following the ADAPT-ITT framework to adapt existing interventions for cultural relevance among groups of people living with HIV, the study team revised an evidence-based intervention, the ‘Stress Management and Relaxation Training/Expressive Supportive Therapy Women’s Project (SMART/EST),’ for online implementation. Working with two community stakeholders, the study team conducted focus groups, theater testing, and manual adaptation. This resulted in the development of e-SMART/EST, an online teletherapy group co-facilitated by a Licensed Psychologist and a credentialed Peer Counselor. The adapted, eight-session weekly intervention was tested with an exploratory pilot sample of eight older women (55 years and older) with HIV and depression. Participants rated the acceptability, feasibility, and appropriateness of the intervention, as well as symptoms of depression and HIV-related quality of life before and after the group. The e-SMART/EST Women’s Project demonstrated high acceptability, feasibility, and appropriateness. Engagement was high, as women attended an average of 6.8 sessions. In qualitative interviews, participants reported peer co-facilitation, culturally relevant themes (e.g., HIV-related minority stress, critical consciousness, grief, and sex and pleasure), mindfulness techniques, and cohesion with other women as main favorable elements of the intervention. Barriers to online implementation included technological issues, distractions due to remote participation, and hindered emotional attunement compared with in-person group therapy. Findings support further research to test similar interventions in full-scale trials with older women living with depression and HIV.

## Women Living with HIV

Women living with HIV (WLWH) have been significantly underrepresented in HIV-related research, often overlooked in the development of pharmacological treatments and clinical services, and marginalized in community-building efforts [1–3]. Initially excluded from AIDS case surveillance in the United Sates, it was not until 1992 that the Centers for Disease Control (CDC) adopted a gender-inclusive definition of AIDS [4]. Subsequently, the number of women identified as living with HIV surged by 151% from 1992 to 1993, marking a pivotal shift in access to treatment and ancillary services for WLWH [5]. More than four decades into the HIV/AIDS epidemic, women now constitute approximately 23.1% of cases in the United States, facing ongoing disparities influenced by racism, sexism, geographical differences in services and resources, and ongoing HIV-related stigma [6, 7]. Despite advancements in antiretroviral treatment, older WLWH continue to encounter chronic stressors such as early-onset menopause and high rates of disability, compounded crises of mental health [8–11]. Older WLWH must content with physiological and sociopolitical stressors [9, 11, 12], which include poverty and low wages, social isolation, gender-based violence, and limited community-based resources dedicated to their needs [13].

## The ’Greying’ of HIV among Women

Globally, over half of people living with HIV (PLWH) are aged 50 and older, a demographic expected to increase to 70% by 2030 [14]. This shift underscores the urgent need to address age-related illnesses, HIV-associated non-AIDS conditions, excess morbidity and mortality, and, notably, mental health concerns [9, 14–16]. The most prevalent psychiatric comorbidity among PLWH is depression, with rates at least double those of the general population [17–19]. Depression is even more common among WLWH than among men [20], and women report both a higher number of symptoms and well as greater symptom severity [21]. These disparities are likely exacerbated by low rates of healthcare engagement and retention among older WLWH [11]. It is critical, therefore, to prioritize older WLWH in mental health research and intervention development.

## Need for Targeted Interventions

Historically, interventions have inadequately addressed the unique needs of older WLWH, who are particularly impacted by internalized societal stigma. There is a need for targeted interventions that center the psychological associations of intersecting stigma related to HIV status, gender, aging, and racial minority status [10, 21]. Myriad studies indicate that older WLWH often internalize negative societal attitudes through processes unique to their experiences as *women* living with HIV [10, 21, 22]. This internalized stigma is strongly associated with elevated rates of depression and lower retention in mental health services, particularly among multiply marginalized WLWH, such as those who are queer and transgender [3, 23], Black and of color [7, 10, 24], and older [9]. To effectively target depression in this growing demographic, interventions must focus on intersecting stigmas as a critical mechanism exacerbating adverse mental health outcomes. Few studies, such as the Women’s Interagency HIV Study (WIHS) [25–27] and the Stress Management and Relaxation Training/Expressive Supportive Therapy (SMART/EST) Women’s Project [28], have made significant strides in measuring and mitigating drivers of health disparities for older WLWH. However, few of these interventions have been made scalable or culturally relevant.

## Developing a Culturally Relevant, Accessible Intervention

**Fig. 1:**
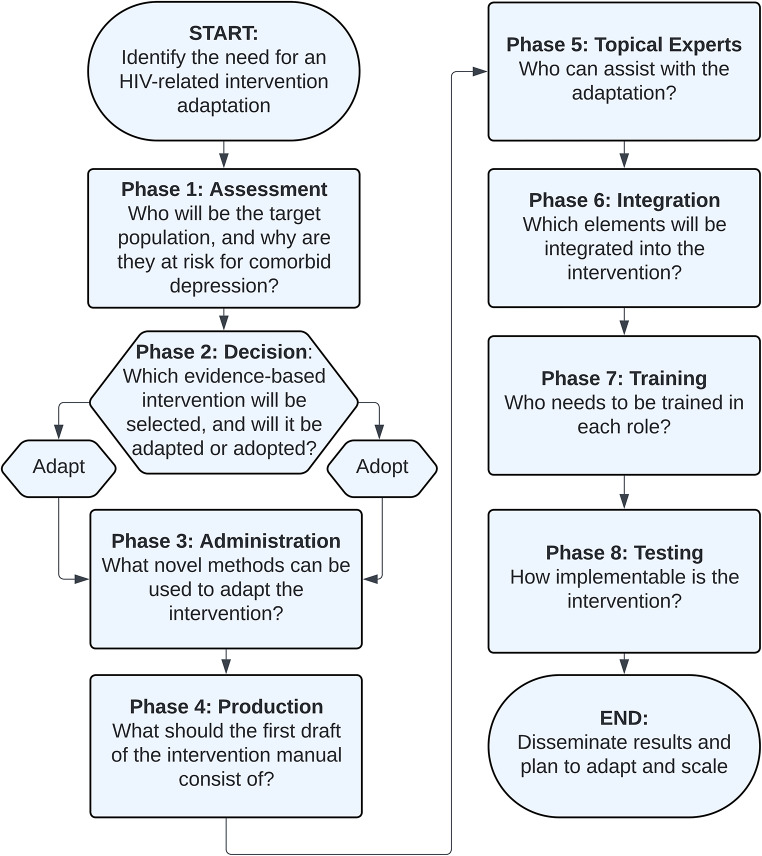
Eight phases and driving questions of the ADAPT-ITT model

In response to these ongoing challenges, this study aimed to adapt the SMART/EST Women’s Project [28] – a previously validated clinical intervention – for an online platform, targeting stigma as a key mechanism of depression. This adaptation process was guided by the ADAPT-ITT model, an eight-phase approach that involves partnering with community experts to ensure cultural and clinical relevance of an adapted intervention [29]. This approach has been successfully applied to tailor interventions for women and for online implementation [30–33]. See Fig. 1 for a visualization of the eight phases and the driving question for each.

In the initial phases of the ADAPT-ITT process, the SMART/EST Women’s Project was selected due to its demonstrated efficacy across diverse groups of women, multiple languages, and various implementation sites over 15 years [28, 34–36]. In its original format, SMART/EST was held in person. Co-facilitators utilized two therapeutic interventions, cognitive behavioral stress management (CBSM+) and expressive-supportive communication, to bolster self-efficacy and shared adaptive coping. The first iteration, SMART/EST I, was implemented in 10 community health centers and yielded significant reductions in depression among WLWH one year post-intervention [36, 37]. SMART/EST II, conducted across 11 health centers in Florida, New York, and New Jersey, consisted of 16 sessions and showed improvements in HIV-related and psychiatric outcomes [34, 38, 39]. Most recently, SMART/EST III was implemented in four HRSA-funded Health Centers, with similarly promising results [40, 41].

To meet evolving needs of older WLWH, the study team aimed to focus on teletherapy to accommodate the increased isolation and service access issues exacerbated by the COVID-19 crisis [42]. Studies from 2020 to 2024 suggest pandemic-related lockdowns may have increased feelings of isolation, fear of coinfection, and heightened depression among older WLWH [42–44]. During this period, there was a significant increase in the utilization of teletherapy—remote audio and video-based mental health services [45]. Teletherapy has proven effective in reducing psychiatric symptoms among diverse groups of PLWH, including older adults [46–48] and those with multiple mental health concerns [43, 49]. Despite drawbacks, such as feelings of emotional distance from providers and technological issues [45], teletherapy offers the potential to integrate mental health services into the ‘daily environment’ and reduce stigma activation common in in-person services [44]. For older WLHW, teletherapy may reduce barriers to service utilization, such as mobility issues, social isolation, and associated costs [50].

## The Present Study

Toward this end, this study aimed to address depression using a therapeutic approach targeting gender-specific processes and internalized stigma among a small pilot group of older WLWH. The present study involved adapting and pilot testing a community-led teletherapy group intervention that centered the expertise of older WLWH. Working with community partners, the study team developed and tested e-SMART/EST, an online version of the SMART/EST Women’s Project. The study team retained aspects of SMART/EST and worked with stakeholders to integrate novel, culturally relevant themes and intervention modalities for the e-SMART/EST teletherapy intervention. The adaptation process harnessed the rapid uptake of teletherapy during the COVID-19 lockdowns, addressing pandemic-related barriers to in-person services. This pilot intervention was co-facilitated via Zoom by a Licensed Psychologist and a Peer Counselor and targeted themes such as minority stress, anger, grief, social support, menopause, sex and pleasure, and harm reduction. The goal was to bolster culturally relevant coping skills such as self-efficacy, mindfulness, social support, and critical consciousness in the context of HIV stigma and intersecting minority stressors [51, 52]. This paper describes two sequential objectives of the study:

1. To utilize the ADAPT-ITT model to adapt the SMART/EST intervention for online implementation as e-SMART/EST.
2. To conduct a small pilot test of the e-SMART/EST intervention and measure its preliminary feasibility, acceptability, and appropriateness for older women contending with the dual impact of HIV and depression.

## Methods and Results of Objective 1: Adaptation

### Adaptation Approach

**Fig. 2:**
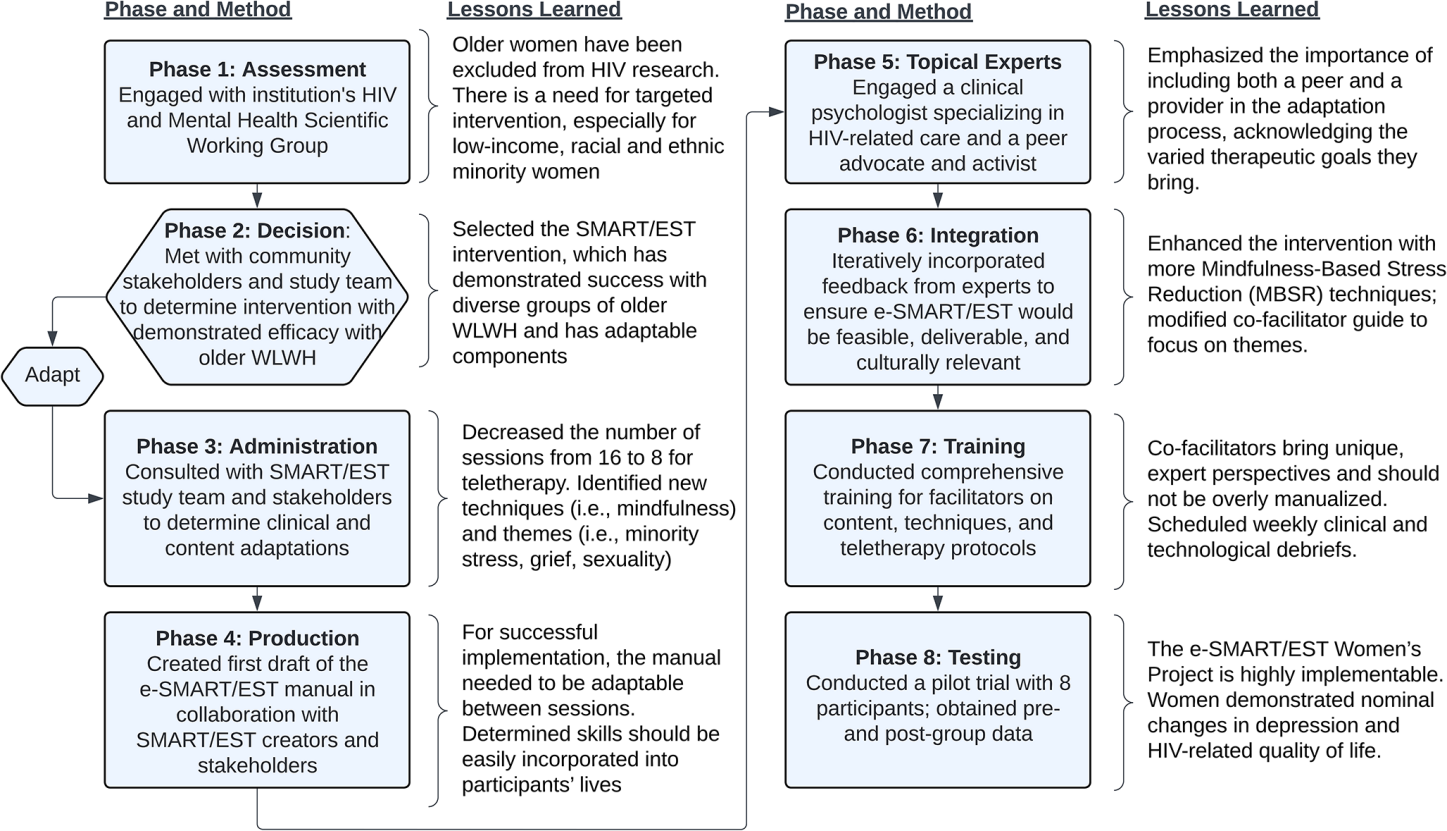
Eight phases, methods, and lessons learned using the ADAPT-ITT model to adapt SMART/EST into e-SMART/EST

The first objective of the study was to adapt SMART/EST into e-SMART/EST. To do so, the study team followed the ADAPT-ITT model, partnering with community stakeholders (i.e., older women with HIV and depression) in each of the eight phases. See Fig. 2 for a summary of the driving question, method, and lessons learned from each phase.

### Adaptation Process and Results

#### Phase 1: Assessment

*Who will be the target population*,* and why are they at risk for comorbid depression?*

In Phase 1, the study team aimed to identify a target population in need of more nuanced intervention to support comorbid HIV and mental health concerns. In 2021, researchers within the Einstein-Rockefeller-City University of New York Center for AIDS Research (ERC-CFAR) launched the HIV and Mental Health Scientific Working Group. This group, composed of basic and translational scientists, clinicians, and prominent community experts (i.e., older adults living with HIV who are involved in research), invested in bolstering mental health for PWLH. During these meetings, two community experts identified older WLWH as a population with marked mental health disparities, namely high rates of depression. Indeed, this population faces unique personal-level stressors (e.g., early-onset menopause, social isolation) and structural-level stressors (e.g., discrimination, stigma, etc.), yet has been the target of limited intervention development and implementation. The study team enlisted the collaboration of members from the Bronx site of the Multicenter AIDS Cohort Study/Women’s Interagency HIV Study Combined Cohort Study (MWCCS) to ensure the project was engaged in ethical practices of women-centered HIV care [53].

#### Phase 2: Decision

*Which evidence-based intervention will be selected*,* and will it be adapted or adopted?*

In phase 2, the study team selected the SMART/EST Women’s Project as the ideal intervention for adaptation. There are limited interventions with empirical support that target mental health comorbidities among WLWH. The SMART/EST intervention, however, has demonstrated efficacy in reducing depressive symptoms, enhancing quality of life, and improving myriad health-related outcomes. While SMART/EST has demonstrated efficacy, it is unclear how feasible the intervention may be via teletherapy. Therefore, the study team decided to adapt, not adopt, SMART/EST and develop the e-SMART/EST teletherapy iteration. The adaptation goals included reducing sessions from 16 to 8, focusing primarily on depression, trialing a teletherapy version, and integrating themes related to minority stressors and intersecting stigma.

#### Phase 3: Administration

*What novel methods can be used to adapt the intervention?*

In Phase 3, we engaged in a pre-testing methodology known as ‘theater testing’ to adapt SMART/EST into e-SMART/EST. The study team met with two focus groups composed of four older WLWH, held online via Zoom. The primary goal of these theater tests was to collect critiques of the existing intervention and identify suggestions for adaptation. Participants were identified through their personal or professional experience working with other WLWH; for example, one participant was a seasoned peer educator for older WLHW. During the theater testing phase, participants were given a description of the intervention, including the goals, session format, and session content/themes. The study team then elicited reactions to study content and materials to assess their reactions and identify recommendations for the additional adaptation, including strengths and drawbacks, topics that were relevant or irrelevant, and overall feasibility, appropriateness, and acceptability. Given the purpose of the trial, the study team inquired about new and existing challenges faced by older WLWH and how the intervention may address such difficulties. Theater testing sessions were recorded and transcribed, and participants were compensated for their time.

Following the focus groups, the research team analyzed the qualitative data to identify themes and recommendations. The results of semi-structured interviews prompted the addition or emphasis of important themes to integrate such as grief and loss, the impacts of race and racism on quality of life, sex and sexuality, anger and assertiveness, and isolation in the age of COVID. The groups emphasized the importance of providing participants with information about HIV-related resources to ameliorate the impact of isolation, and recommended utilizing multiple therapeutic modalities such as mindfulness in addition to psychoeducation.

#### Phase 4: Production

*What should the first draft of the intervention manual consist of?*

In Phase 4, the study team created a first draft of the adapted e-SMART/EST manual. To adapt rather than adopt, the study team retained the framework and core content of the SMART/EST Women’s Project, with some content and timeline amendments made to better address concerns specific to older WLWH. In line with feedback from focus group members provided during theater testing in Phase 3, we condensed the intervention from 16 to 8 sessions based on concerns that longer trials were burdensome for older adults. In the Production phase, we created an initial draft of the e-SMART/EST manual with eight sessions, including some that incorporated new themes suggested by focus group members: aging, anger, and assertiveness for older women of color (Session 3); grief, loss, and coping (Session 4); and dating, sex, and love (Session 7). Practical recommendations from the focus groups, such as holding sessions after 5pm and making reminder calls, were integrated to enhance accessibility. Further, we incorporated on-call technological support and Zoom training given some participants may not be familiar with using videoconference software.

#### Phase 5: Topical Experts

*Who can assist with the adaptation?*

In Phase 5, we enlisted the topical expertise of a Licensed Psychologist who conducted SMART/EST III to inform further adaptation and co-facilitate the pilot group alongside our second topical expert, a Peer Counselor with decades of experience supporting older WLWH. The Psychologist recommended incorporating mindfulness-based stress reduction (MBSR) techniques and recommended incorporating these techniques to complement SMART/EST’s utilization of CBSM + and expressive-supportive communication. Specifically, this expert adapted the manual to include somatic stress-reduction practices with demonstrated efficacy among WLWH [54, 55]. The addition of MBSR was ideal for the adapted intervention, given its utility in reducing proximal minority stressors and improving self-efficacy, for example, among sexual minority people [56] and young sexual and gender minority people of color [57]. We thus incorporated breathing exercises, body awareness meditations, the emotional freedom technique (colloquially referred to as ‘tapping’), and guided meditations.

#### Phase 6: Integration

*Which elements will be integrated into the intervention?*

During the Integration phase, we integrated feedback from our theater testing focus groups and meetings with topical experts to produce a second draft. This consisted of determining which elements of SMART/EST to retain and which to adapt. This penultimate manual outlined clinical practices for eight weekly 90-minute sessions, with content focused on themes with clinical and sociopolitical relevance for older WLWH. Sessions, outlined in Table 1, centered on the following themes: *Session 1*, Introduction to the impact of stress on physical and mental health; *Session 2*, Linking thoughts, feelings, and behavior in the context of minority stress; *Session 3*, Aging, anger and assertiveness; *Session 4*, Grief, loss, and coping; *Session 5*, Guilt, self-care, and social support; *Session 6*, Self-esteem, menopause, and loving your body; *Session 7*, The joy of sex and pleasure; and *Session 8*, Review, harm reduction, and looking forward. Each session followed a similar format, with four sequential components: (1) introduction and check in; (2) psychoeducation and process; (3) putting it into practice; and (4) invitation for the week ahead.

**Table 1:** Flow and session content of the e-SMART/EST women’s project

*Session Flow*			
**Introduction and Check-In** **(first 15 min)**	**Psychoeducation and Process** **(30 min)**	**Putting it into Practice** **(30 min)**	**Invitation for the Week Ahead** **(final 15 min)**
**Session 1**: Introduction to the impact of stress on physical and mental health Subthemes: group rapport; HIV stigma; depression			
Introduce women to the group. Co-create group guidelines and goals. Troubleshoot technical issues. Lead grounding exercise.	Discuss the relationship between stressors, stress responses, immune system, and depression. Begin to introduce CBSM + and MBSR techniques.	Introduce guided meditation. Lead group in emotional freedom technique (i.e., ‘tapping’ exercise), then process somatic, cognitive, and emotional experiences during the exercise.	Access meditation app to engage in grounding meditation about safety amidst stigma; practice ‘tapping’ exercise
**Session 2**: Linking thoughts, feelings, and behavior in the context of minority stress Subthemes: HIV-related stress; aging and discrimination; combating internalized stigma			
Lead grounding exercise. Review group guidelines and theoretical model. Discuss examples of stressors and their impact on thoughts, feelings, and behavior	Share slides describing thoughts-feelings-behavior triangle. Discuss mind-body connection. Discuss reactions to MBSR techniques, including barriers and hope for recovery.	Lead group in ‘slowing your mind’ meditation, then discuss somatic, cognitive, and emotional experiences during the meditation.	Access mediation app to engage in ‘slowing your mind’ meditation. Be mindful of thoughts that reinforce stigma
**Session 3**: Aging, anger, and assertiveness Subthemes: righteous anger; politics of gender, race, and anger; assertiveness			
Lead grounding exercise. Review thoughts-feelings-behaviors triangle. Discuss examples that came up between sessions.	Share slides describing minority stress and intersecting systems of oppression. Introduce concept of critical consciousness. Discuss the impact of anger on the body	Lead group in ‘letting go of anger’ meditation, then discuss somatic, cognitive, and emotional experiences during the meditation.	Access meditation app to engage in ‘letting go of anger’ meditation. Practicing feeling the anger, not ‘becoming’ the anger. Practice assertiveness.
**Session 4**: Grief, loss, and coping Subthemes: aging after loss; HIV-related survivor’s guilt; coping with grief			
Lead grounding exercise. Review anger as a political process and minority stress content. Introduce group to grief and coping.	Share slides describing links between anger and sadness in the context of grief. Introduce compassion and social support as antidotes to grief.	Co-create suggestions for strategies to cope with grief. Lead group in ‘grief and loss’ meditation and process somatic, cognitive, and emotional experiences during the meditation.	Access meditation app to engage in ‘grief and loss’ meditation. Reconnect with someone you have been missing.
**Session 5**. Guilt, self-care, and social support Subthemes: self-care; self-efficacy; guilt; community resources and support			
Lead grounding exercise. Review grief, loss, and coping content. Introduce need for further social support.	Share slides about self-care in the context of poverty, HIV survivorship, and expectations of older women of color.	Co-generate list of racial-culturally appropriate self-care rituals. Discuss relational barriers to self-care.	Practice behavioral activation for self-care. Write a list of community resources and engage 2–3 sources of support.
**Session 6**: Self-esteem, menopause, and loving your body Subthemes: self-efficacy; self-empowerment; managing menopause			
Lead grounding exercise. Review content about barriers to self-care and need for community resources. Introduce links between menopause, HIV, and depression	Share slides about menopause and aging with HIV. Discuss the impact of long-term ART use on somatic experiences. Introduce bodily self-compassion.	Lead ritual of bodily self-care. Lead group in ‘body positivity’ meditation and process somatic, cognitive, and emotional experiences during the meditation.	Access meditation app to engage in ‘body positivity’ meditation. Reach out again to 2–3 sources of social and community support.
**Session 7**: The joy of sex and pleasure Subthemes: HIV-related quality of life; sex and pleasure			
Lead grounding exercise. Review menopause content. Discuss impact of aging on sex and pleasure.	Lead group in ‘the joys of sex and pleasure’ slides to empower women to reclaim sexuality and pleasure. Discuss trauma related to sex, side effects of ART, and HIV status disclosure.	Lead ritual in ‘reclaiming your pleasure’ meditation, then discuss somatic, cognitive, and emotional strategies to make room for pleasure.	Access meditation app to engage in ‘reclaiming your pleasure’ meditation. Engage in ritual of self-pleasure and/or bodily empowerment.
**Session 8**: Review, harm reduction, and looking forward Subthemes: emotional vulnerability; social connection; maintaining mental wellness			
Lead grounding exercise. Lead group in review of past seven sessions.	Lead group in review of the coping skills, mindfulness techniques, and cognitive-behavioral strategies.	Lead emotionally expressive group process about the past two months together to reinforce social support.	Encourage self-reinforcement of skills, principals, and engagement with community and social support.

#### Phase 7: Training

*Who needs to be trained in each role?*

In Phase 7, the study team met with co-facilitators to plan for e-SMART/EST implementation. We met for multiple training sessions, during which the co-facilitators participated in supervision and mock sessions, providing bidirectional feedback. These training sessions provided an overview of the updated manual and allowed co-facilitators the time and opportunity to internalize the structure of the manual and make changes to session content. Following the initial training, the study team identified an ongoing training schedule in which the primary investigator and co-facilitators met for weekly advisory sessions. This group supervision involved a pre-group training refresher (in which co-facilitators discussed the content and then self-delegated tasks for the upcoming session), and a post-group debrief (in which the primary investigator elicited feedback and provided clinical support to co-facilitators).

#### Phase 8: Testing

*How implementable is the intervention?*

After completing phases 1–7, the study team established a protocol for online implementation via Zoom, and provided a pre-group ‘Zoom training’ for participants. We then recruited eight women and administered a pre-group survey, followed by eight sessions of the adapted intervention and a post-group survey. Sessions were recorded, transcribed, and de-identified for supervision, additional training, and further iterations of manual adaptation. The testing phase allowed us to evaluate the acceptability, feasibility, and appropriateness of e-SMART/EST. In the post-group survey, participants completed repeat measures as well as a mixed-methods survey to elicit feedback about the intervention’s content, methods, co-facilitation, and online delivery.

## Methods and Results of Objective 2: Pilot Testing

### Participant Eligibility and Recruitment

At the end of the ADAPT-ITT process, we pilot tested e-SMART/EST, a teletherapy trial with eight women 55 years and older living with HIV and experiencing past-year depressive symptoms. Recruitment was led by a Research Coordinator, who partnered with the Bronx site of MWCCS [16] to identify, contact, and screen women living with comorbid HIV and past-year depression. Seventy-one women were identified based on past-year positive screens on the Center for Epidemiological Studies-Depression screener (CES-D score ≥ 16) [58]. We contacted a random selection of 17 women initially, of whom 10 were eligible and consent to participate. Five of the 17 were screened out because they were not interested, and two were screened out due to having moderate to severe cognitive impairment (detected via a score ≥ 12 on the Telephone version of the Montreal Cognitive Assessment, or T-MoCA) [59]. While 10 women were screened and determined eligible, two additional participants dropped out before the group, resulting in a final sample of eight women.

Study procedures consisted of 10 remote visits. The first was a pre-group survey, administered live via Zoom, capturing sociodemographic characteristics, depression symptomatology, and HIV-related quality of life. Participants then completed eight weekly group sessions held via Zoom. Following the eighth session, they completed a post-group survey held via Zoom, completing repeated measures and providing reactions and recommendations to the group. All procedures were approved by the Institutional Review Board at Albert Einstein College of Medicine, Protocol No. 2021–13,391.

### Measures

#### Sociodemographic Characteristics

During the pre-group survey, participants self-reported race and ethnicity, gender identity, sexual orientation, education level, annual income, sources of social support, age; and health-related characteristics including menstrual status, lifetime history of AIDS diagnosis, viral load at last visit, and years since diagnosed with HIV.

#### Engagement

Levels of engagement were measured by averaging the number of sessions participants attended on average, of eight, as well as the number of attendees per session.

#### Fidelity

To capture fidelity, the PI coded each session in terms of how closely co-facilitators adhered to the tenets and therapeutic approach of the e-SMART/EST manual. We created an adapted fidelity checklist based on a validated checklist from Project AFFIRM, a minority stress intervention for sexual and gender minority adults [60]. Each session was coded using eight indicators of fidelity, detailed in Table 3. Each indicator was scored from 0 to 3, for a total session score of 24, with higher scores indicating higher fidelity. To improve fidelity, the PI scored each session after it was completed, then met with co-facilitators for clinical supervision. During these meetings, the study team make manual adjustments for the upcoming session and processed group dynamics, co-facilitation dynamics, and emergent themes.

#### Feasibility, Acceptability, and Appropriateness

To measure how implementable e-SMART/EST was, we used validated scales to capture three outcomes: feasibility, or how accessible and easy it was to engage in the group; acceptability, or how satisfying and enjoyable e-SMART/EST was to participate in; and appropriateness, or how culturally and clinically relevant e-SMART/EST was for older WLWH. These were captured during the post-group survey via three Likert-type scales: the Feasibility of Intervention Measure (FIM); Acceptability of Intervention Measure (AIM); and Intervention Appropriateness Measure (IAM) [61]. Each scale had four items with response options ranging from 1, completely disagree, to 5, completely agree. The measure uses am established score of **‘**3’ to indicate neutral responses from participants, with higher scores indicating positive feelings; thus, we used an average score higher than ‘3’ to indicate successful implementation. We also captured participants’ perceptions of feasibility, acceptability, and appropriateness via brief, semi-structured interview conducted via Zoom during the post-group survey. See Table 4 for a joint display, as well as the interview questions and demonstrative quotes for each implementation outcome.

#### Depression and HIV-related Quality of life

To capture preliminary efficacy, we collected repeat-measures data about the pilot group’s levels of depression symptoms and HIV-related quality of life. During the pre- and post-group surveys, participants completed the 21-item Beck Depression Inventory (BDI) [62] and the 12-item, condensed version of the Medical Outcomes Study-HIV Health Survey (MOS-HIV SF-12) [63]. The BDI is one of the most utilized scales to measure depression symptomatology, with scores between 0 and 13 indicating minimal depression, 14–19 mild, 20–28 moderate, and 29–63 severe. The MOS-HIV SF-12 is a commonly used scale to capture HIV-related quality of life, with higher scores indicating better outcomes.

### Statistical Analysis

To assess implementation outcomes, the study team calculated mean scores for feasibility (FIM), acceptability (AIM), and appropriateness (IAM), along with session fidelity and overall fidelity across eight sessions. Scores of 3 or higher were considered reflective of successful implementation. Engagement was determined by averaging participants’ attendance scores.

For qualitative data, thematic analysis was conducted by the study team. We applied implementation construct codes (i.e., acceptability, feasibility, appropriateness) to match qualitative data with quantitative outcomes. We conducted individual, semi-structured interviews with each participant who completed the group. See Table 4 for the questions asked during individual interviews. Qualitative data were analyzed using an inductive thematic approach with three a priori themes: acceptability, feasibility, and appropriateness. The coding process was led by two members of the study team, who applied these a priori themes to qualitative data. They first applied themes separately, then met to resolve discrepancies.

To integrate quantitative with qualitative data, we used a program evaluation method and a joint display (Table 4) to report integrated results. In exploratory analyses, preliminary efficacy was evaluated with paired dependent t-tests to compare changes in depression (via the BDI) and HIV-related quality of life (MOS-HIV SF-12). We calculated mean differences, t-statistics, and significance (α < 0.05) for these outcomes, with analyses performed using SPSS.

## Results of the e-SMART/EST Pilot Test

### Sample Characteristics

During the testing phase, we conducted an exploratory trial of e-SMART/EST with a pilot sample of eight women. All women identified as women of color, with majority being African/African American/Black (*n* = 5, 62.5%), followed by Hispanic/Latina/o American (*n* = 3, 37.5%), then American Indian/Native American (*n* = 1, 12.5%). Though the group was open to cisgender and transgender women, all participants identified as cisgender. Six of the eight women (75%) were heterosexual/straight, one was bisexual, and one was lesbian. Education level ranged from ‘did not finish high school/GED’ (*n* = 2, 25%) to ‘finished graduate school’ (*n* = 2, 25%), though most participants completed at least some trade school or college. The majority (*n* = 7, 85.5%) earned between $0-$60,000 whereas one participant (who was employed full-time with an HIV service organization) made between $60,000-$80,000. In terms of social support, five women (625.5%) reported ‘other women living with HIV’ as a primary source of support; other sources included family (*n* = 4, 50%), clinical team (*n* = 1, 12.5%), and other (*n* = 4, 50%). When screened, three women (37.5%) had no cognitive impairment, while five (62.5%) were shown to have mild cognitive impairment. Seven of eight women were post-menopausal, while one woman had never menstruated. The mean age was 59.5 years old. In terms of HIV-related characteristics, half reported they had been diagnosed with AIDS during their lifetime; the majority (*n* = 7, 87.5%) had an undetectable viral load at their last visit; and the pilot group had been living with HIV for an average of 29.1 years. See Table 2.

**Table 2:** Characteristics of the pilot sample (*n* = 8)

	*n*	%
Race and Ethnicity		
African/African American/Black	5	62.5%
American Indian/Native American	1	12.5%
Hispanic/Latina/o American	3	37.5%
Cisgender/Non-Transgender Woman	8	100.0%
Sexual Orientation		
Bisexual	1	12.5%
Heterosexual/Straight	6	75.0%
Lesbian/Gay	1	12.5%
Education Level		
Did not finish high school/GED	2	25.0%
Some college	1	12.5%
2-year college degree	1	13.0%
4-year college degree	1	13.0%
Trade school	1	13.0%
Finished graduate school	2	25.0%
Annual Income		
$0-$60,000	7	87.5%
$60,001-$80,000	1	12.5%
Sources of Social Support		
Family	4	50.0%
Clinical team	1	12.5%
Other women living with HIV	5	62.5%
Other	4	50.0%
Age [Mdn (IQR), SD]	59.5 (4.5), 3.5	
Post-Menopausal	7	87.5%
Ever diagnosed with AIDS	4	50%
Undetectable viral load at last visit	7	87.5%
Years since HIV diagnosis [M (SD)]	29.1 (9.3)	

*Note* Mdn: median; IQR: interquartile range; SD: standard deviation; M: mean

### Implementation Outcomes

Participant feedback about the group was highly favorable overall. All participants recommended expanding the intervention to include larger groups of older women with comorbid HIV and depression, indicating successful implementation.

**Engagement.** Average weekly attendance was 83% on average, with participants attending 6.38 of eight sessions on average. In qualitative interviews, participants generally indicated a desire for more, opposed to fewer, sessions. One participant (pseudonym ‘Lori,’ 59) said, “What was it, eight weeks? It wasn’t really enough. I would have [shared] more if we would’ve had more time.” Participants reported issues to engagement such as technical difficulties, scheduling conflicts, and health issues (e.g., feeling too tired to log on, or a doctor’s appointment running over time).

**Fidelity (**Table 3**)**. Co-facilitators were highly adherent to the e-SMART/EST manual, with an average of 90% fidelity across sessions. Fidelity ranged from 75% in Session 8 to 100% in Sessions 2 and 3.

**Table 3:** e-SMART/EST women’s project fidelity checklist

Fidelity Indicator	Session Number								Fidelity Score (24 Total)	% Fidelity
	1	2	3	4	5	6	7	8		
1. Delivers the e-SMART/EST intervention as intended	2	3	3	2	3	2	3	2	20	83.33%
2. Frames depression among older WLWH in a socio-ecological context (i.e., discusses HIV stigma, minority stress, and empowerment)	3	3	3	3	3	3	3	3	24	100.00%
3. Presents psychoeducation material on factors associated with depression among older WLWH	3	3	3	3	3	3	3	3	24	100.00%
4. Enhances participants’ knowledge about adaptive coping strategies	3	3	3	3	3	3	3	3	24	100.00%
5. Effectively uses CBSM + interventions to empower participants to respond in adaptive ways to HIV-related stressors	3	3	3	3	1	2	1	2	18	75.00%
6. Effectively uses MBSR techniques to empower participants to notice and reduce bodily and emotional manifestations of depression and stress	3	3	3	3	3	3	3	3	24	100.00%
7. Global session rating	2	3	3	3	1	3	1	1	17	70.83%
8. Effectively co-facilitates session (i.e., integrates themes, attends to all participants)	3	3	3	3	3	3	3	1	22	91.67%
**Total Score Per Session (out of 24)**:	22	24	24	23	20	22	20	18		
**Percent fidelity per session**	91.67%	100.00%	100.00%	95.83%	83.33%	91.67%	83.33%	75.00%	**Overall fidelity**:	90.10%

*Note* e-SMART/EST: Online iteration of the Stress Management and Relaxation Training/Expressive Supportive Therapy Women’s Project; WLWH: women living with HIV; CBSM+: cognitive-behavioral stress management with expressive-supportive communication; MBSR: mindfulness-based stress reduction

**Feasibility (**Table 4**).** Participants found the e-SMART/EST intervention highly feasible, suggesting it was accessible and easy to engage in (FIM mean = 4.22). ‘Didi’ (58) expressed: “I looked forward to it. I didn’t miss a group.” When asked about the number of sessions and time commitment, participants denied concerns about treatment burden. ‘Marlene’ (58) shared: “I make sure that my schedule was clear.” ‘Desiree’ (61) commented: “It needed to be every week […] it allows you to build relationship consistency across the board with the other women.” Those who were employed reported the online modality increased their ability to attend. Some women, however, faced feasibility challenges due to the online format. ‘Lori’ (59), for example, lost track of the calendar invitation and was unsure how to log onto Zoom: “I couldn’t get in, and I had nothing to do, so I was very discouraged.” Overall, women reported the content was easy to engage in, largely due to the slides and the co-facilitators explaining complex phenomena in approachable language.

**Acceptability (**Table 4**).** Participants also found the intervention to be highly acceptable (AIM mean = 4.06), suggesting it was satisfying, enjoyable, and easy to engage in. Highlights identified in qualitative interviews included the sense of community, peer co-facilitation, and the MBSR techniques. ‘Dawn’ (63) noted the sense of comradery in the group: “I enjoyed the group immensely. Interacting with the ladies, being able to be personal with [the facilitators]. We’re all on an even keel.” Many women were struck by the invitation for open dialogue about HIV and intersectional issues. As ‘Paula’ (67) said: “I love the group. It gives me a chance to open up about things that I’ve been going through that I don’t have a chance to speak [about] with other people.” ‘Desiree’ noted: “I like attending group more than going to the doctor […] that’s why I go to group. To learn, interact, build community.” Key aspects enhancing acceptability included: peer co-facilitation (e.g., “It needs to be a peer facilitator there,” ‘Desiree,’ 61) and mindfulness exercises (e.g., “[I enjoyed] the meditative part of the group, doing the meditation, letting go,” ‘Dawn,’ 63).

Another highlight was the chance for expressive emotional communication as a counterbalance to shame related to HIV, aging, and menopause. ‘Paula’ (67) described: “I really enjoyed it because it gave me a chance to speak my feelings where I don’t have to feel ashamed.” ‘Marlene’ (58) agreed: “With COVID and depression […] we do need an outlet. We need someone, some place to talk, to hear other people’s stories and ways to cope.” As ‘Didi’ (58) said, “[The group] helped me connect with others who have the virus living through different circumstances. […] People do need people to talk to. They need somebody to reach out to […] We all got same issues.” Expressive emotional communication was especially helpful for contending with minority stressors,. ‘Lori’ (59) said: “It helped me to think about some of the things us females go through […] if you listen, you’ll learn from others […] and it’ll probably take you a little further.”

**Appropriateness (**Table 4**).** Last, the intervention was highly appropriate (IAM mean = 4.25), suggesting it was culturally and clinically relevant for older WLWH. Participants found the session themes, peer-based model, and group dialogue especially relevant. ‘Desiree’ (61) noted: “I thought the topics were really relevant to women of color. There’s additional stressors […] African Americans endure just because we’re Black people living in this country […] so I think that was real relevant.” The co-facilitation format was seen as crucial to ensuring older WLWH found the group to be relevant. ‘Marlene’ (58) said: “They work hand-in-hand with each other, and I like that, and I really enjoyed that. They had a good bond, and I noticed the chemistry was very good.” Having an out and proud peer co-facilitating the group balanced perceived mistrust or hesitancy and ensured participants felt the group was made ‘for us, by us.’ As ‘Didi’ (58) said:

*If [the peer facilitator] wasn’t there*,* then how would [the Psychologist] be able to understand that it could be a stigma? A lot of women […] expressed what they was going through. She was facilitating the group and yeah*,* she was doing a very good job.*

Key session topics such as menopause, anger, and grief increased perceived appropriateness of the group. Multiple participants recommended additional themes such as motherhood and family building to increase the relevance for other older women.

**Preliminary efficacy**. Though results of the pre-post-group t-tests were nonsignificant, mean depression scores decreased nominally from 16.0 (SD = 7.7) to 13.88 (SD = 8.9). HIV-related quality of life (MOS-HIV SF-12v2) showed nominal increase from 79.9 (SD = 15.5) to 80.5 (SD = 14.2).

**Table 4:** Joint display describing feasibility, acceptability, and appropriateness of the e-SMART/EST women’s project

Quantitative Measures			Qualitative Measures	
Scale	Mean (SD)	% above cutoff	Qualitative interview questions	Demonstrative quote
Feasibility: How accessible and easy is it to engage in e-SMART/EST?				
Feasibility of Intervention Measure (FIM)	4.22 (0.91)	90.60%	1. How was it for you to attend once a week? Was it too much? Too little? 2. What made it easier or more difficult for you to attend sessions each week?	“I make sure that Thursdays I’m clear. I have to be there, you know? I’m not getting any other support from anywhere else and having that group was just like, ‘Wow!’ In a time like what’s going on with COVID and depression and all those stuff going around, you know, we do need an outlet. I make sure that my schedule was clear.” (‘Marlene,’ 58)
Acceptability: How satisfying, enjoyable is e-SMART/EST?				
Acceptability of Intervention Measure (AIM)	4.06 (1.25)	75.00%	1. To what extend did you enjoy or not enjoy the group? 2. How do you feel about working with a peer facilitator?	“I love the group. It gives me a chance to open up about things that I’ve been going through that I don’t have a chance to speak with other people, because I’m on a need-to-know basis and being that I’m sorta closed about the, you know, discussing my HIV status with anybody. It was fun. It was good being in that group for me.” (‘Paula,’ 67)
Appropriateness: How culturally and clinically relevant is e-SMART/EST for older WLWH?				
Intervention Appropriateness Measure (IAM)	4.25 (0.8)	93.80%	1. Would you recommend this group to a friend, another woman living with HIV? 2. What are some topics that you wish we had covered? 3. How might the group have helped you understand your issues or concerns better?	“I thought the topics were really relevant to women of color or women, women… well, women of color, because they did have that segment around ‘Breathing While Black.’ I also think the rest of the intervention really spoke to the aging population of HIV positives.” (‘Desiree,’ 61)

## Discussion

Preliminary results from the e-SMART/EST pilot underscore the need for accessible, culturally relevant group interventions for older women with HIV and comorbid depression. Given high ratings of acceptability, results suggest participants valued e-SMART/EST as a vital resource to address issues including complex grief, stigma, and social isolation associated with aging with HIV. While we faced challenges related to technological literacy, privacy concerns, and emotional distancing due to the online format, the group positively received the content, peer co-facilitation, and the emphasis on resilience and mindfulness. We thus offer key lessons learned for future interventions promoting holistic health for older WLWH.

First, much of the success of the pilot intervention was due to its women-centered, community-participatory approach to HIV research and care [2, 53], [64]. From conceptualization to adaptation to pilot testing, we centered the needs and expertise of older WLWH. In early meetings, our theater testing group identified the objectives of the study, encouraging a meaningful intervention to target depression among older WLWH. Rather than extract their expertise, we hired experts into paid leadership roles through which they led the adaptation process using the ADAPT-ITT model [29]. This process was clinically and socio-politically meaningful for all involved. Most critically, the inclusion of a trained peer co-facilitator as a co-facilitator was a key ingredient for the group and was critical to building trust, rapport, and genuine engagement.

Second, we recommend future interventions with older WLWH incorporate culturally adapted mindfulness-based techniques. Each session of e-SMART/EST concluded with a guided meditation or related mindfulness intervention, which participants described favorably as a key tool to target the cognitive, emotional, and somatic sequala of living with HIV and depression. Based on recommendations from studies with Black and other women of color living with HIV [54, 55], co-facilitators chose meditations led by older women of color that explicitly addressed the impact of sexism, racism, and aging on women’s bodies and holistic health. The acceptability of MBSR interventions suggest further evidence for their utility to improve self-efficacy among PLWH [65].

Third, e-SMART/EST addressed the processes by which older WLWH internalize intersectional stigmas related to HIV, racism, sexism, and ageism. Co-facilitators, for example, provided psychoeducation on structural determinants of mental health and led interventions to foster critical consciousness and empowerment. This focus enabled participants to locate the source of distress within a societal context, rather than focusing myopically on individual or intrapsychic drivers. We recommend a similar focus in future interventions, particularly given the proliferation of stigma and disempowerment among older WLWH [51], [66].

Finally, while the use of teletherapy may increase access for older populations, it also presents specific challenges for older WLWH. The varying technological support needs of participants, ranging from none to weekly assistance, imposed demands and raised equity concerns [31]. Further, participants appreciated how easy it was to attend the group from home, yet also reported yearning for in-person connection after the pilot ended. While teletherapy should continue to be a focus for the initial engagement of diverse older adults in research and care, it may be best followed by hybrid programming (i.e., accessible via in-person and online formats) to better meet evolving needs. Despite drawbacks, teletherapy offers significant benefits, particularly for marginalized groups such as older WLWH who have high rates of disability, immobility, and social isolation [50].

## Limitations

This pilot study had notable limitations. First, it was conducted with a small sample and a single test, lacking the statistical power necessary for broader conclusions and highlighting the need for larger-scale, randomized-controlled trials to evaluate the intervention’s efficacy more rigorously. Despite this small sample, this study contributed to the literature by providing a more culturally-relevant adaptation of the ‘SMART/EST Women’s Project,’ including information and feedback from key stakeholders. Results also suggest that this psychotherapy group was feasible for this small sample of older WLWH; thus, future research my choose to replicate this project with a larger sample size. Indeed, our pilot sample of eight women was very small. The study team does not intend to draw broader conclusions about the generalizability of the current study nor the mental health of older WLWH in general from this very small pilot sample. Second, the absence of a control or waitlist condition in this pilot indicates the importance of such designs in future research. Additionally, participants were recruited from the MWCCS study, which may have introduced sampling bias as these women were already engaged in HIV-related research. Findings from the study thus may not be generalizable to less research-engaged older WLWH. Third, data collection occurred shortly after the pilot without a long-term follow-up, and thus there may have been a ‘halo effect’ whereby participants rated the intervention as successful due to its recent implementation. Future full-trial iterations would ideally include longitudinal follow-up and extended assessments periods to more fully measure implementation outcomes and preliminary efficacy. Fourth, while qualitative interviews queried about acceptability, feasibility, and appropriateness, the study team did not elicit further phenomenological detail about our participants’ HIV-related quality of life. Such questions would have deepened our understanding of the impact of aging with HIV among this pilot group and, as such, should be central to future iterations of this line of research. Finally, technological challenges, including connectivity issues and the distractions of remote participation, were encountered. These limited the ease of feasibility, yet also provided valuable lessons for refining the e-SMART/EST and similar teletherapy groups with older WLWH.

## Conclusions

Despite its limitations, this study underscores the potential of teletherapy for older WLWH: a highly resilient group facing intersecting social determinants of mental health. Future research should involve larger, more diverse samples to comprehensively evaluate the intervention’s effectiveness on mental and physical health outcomes. Demonstrating efficacy in larger studies, particularly regarding depression and quality of life, could position this group as a key modality for accessible mental health services with this growing demographic.
